# Synergistic effects of plant defense elicitors and *Trichoderma harzianum* on enhanced induction of antioxidant defense system in tomato against Fusarium wilt disease

**DOI:** 10.1186/s40529-017-0198-2

**Published:** 2017-11-02

**Authors:** Andleeb Zehra, Mukesh Meena, Manish Kumar Dubey, Mohd. Aamir, R. S. Upadhyay

**Affiliations:** 1 Laboratory of Mycopathology and Microbial Technology, Centre of Advanced Study in Botany, Banaras Hindu University, Varanasi, Uttar Pradesh 221 005 India; 2 Department of Botany, Banaras Hindu University, Varanasi, 221 005 India

**Keywords:** *Trichoderma harzianum*, Chemical inducers, Reactive oxygen species, Antioxidant system, Lipid peroxidation

## Abstract

Plant defense against their pathogens can be induced by a complex network of different inducers. The present study investigates the synergistic effect of *Trichoderma harzianum*, exogenous salicylic acid (SA) and methyl jasmonate (MeJA) over the response and regulation of the antioxidant defense mechanisms and lipid peroxidation in tomato plants against Fusarium wilt disease. In the present work, tomato plants were infected by *Fusarium oxysporum* f. sp. *lycopersici* 3 days after inoculated with *T. harzianum* and/or sprayed daily for 3 days with chemical inducers (SA and MeJA). Plants were analysed at 0, 24, 48, 72 and 96 h after inoculation with *Fusarium oxysporum* f. sp. *lycopersici*. Infection of tomato plants by pathogen led to strong reduction in the dry weight of roots and shoots with the enhanced concentration of H_2_O_2_ and varying degree of lipid peroxidation. Concurrently, exogenous SA, when applied with pathogen greatly enhanced H_2_O_2_ content as well as activities of antioxidant enzymes except catalase (CAT) and ascorbate peroxidase (APx). The pathogen challenged plants pretreated with *T. harzianum* and MeJA together exhibited less lipid peroxidation and as well as the elevated level of ascorbic acid and enhanced activities of antioxidant enzymes. All applied treatments protected tomato seedlings against Fusarium wilt disease but the percentage of protection was found higher in plants pretreated with the combination of *T. harzianum* and chemical inducers.

## Introduction

Tomato (*Lycopersicon esculentum* Mill.) is a major contributor to the fruits and vegetable diet of humans throughout the world (Kapsiya et al. 2015). However, its growth, yield and economic productivity are significantly reduced by several pathogens. Fusarium wilt disease of tomato caused by *Fusarium oxysporum* f. sp. *lycopersici* is one of the most devastating disease worldwide which causes yield reduction and affects tomato production (Hanaa et al. 2011). The fungal pathogen resides in the soil and causes infection to susceptible tomato plants by the roots throughout their course of development causing necrosis and inducing wilting of the plants at the later stages of their infection and reduces the yield and hence limits the overall economic productivity (El-khallal 2007a, b).

Many biotic and abiotic agents can effectively induce plant resistance against various pathogens (Ramamoorthy et al. 2001; Edreva 2004). Bio-agents can induce resistance against various diseases caused by many pathogens such as viruses (Maurhofer et al. 1994), bacteria (Park and Kloepper 2000) and fungi (Bokhari and Perveen 2012). In spite of these, some bioagents such as *Trichoderma* spp. have been reported to promote plant growth, nutrient uptake, and induction of plant defense responses against different biotic and abiotic stresses (Harman et al. 2004; Shoresh et al. 2010; Hermosa et al. 2012). There are several evidences which support that *Trichoderma* spp. are able to induce the defense mechanisms in several plants (Vinale et al. 2008; Brotman et al. 2012). The results obtained through 2D electrophoresis and high-density oligonucleotide microarrays confirmed the induction of proteome and differential gene expression in plant system when inoculated with *Trichoderma* spp. (Marra et al. 2006; Alfano et al. 2007; Segarra et al. 2007). Different chemical inducers such as salicylic acid (SA), jasmonic acid (JA) and methyl jasmonate (MeJA) are signal molecules that play a key role in plant growth and development, and in the induction of plant defense responses to various abiotic and biotic stress factors (Sticher et al. 1997; War et al. 2011a, b). Various physiological, biochemical and molecular processes in plants including antioxidative enzyme activities can be manipulated by exogenous application of SA and JA (Idrees et al. 2011; War et al. 2011a, b). SA and MeJA play a role in various signal transduction mechanisms associated with plant defense and also elicit the expression of various important enzymes catalyzing a wide array of biosynthetic reactions whose products, through a complex network results in formation of various types of defense compounds including polyphenols, alkaloids and pathogenesis-related (PR) proteins (Hahlbrock and Scheel 1989; Creelman and Mullet 1995), conferring plant protection from pathogen-attack (Delaney et al. 1994; Kozlowski et al. 1999). The transcriptional reprogramming associated with plant defense responses are mediated by SA against different biotic and abiotic stresses (Herrera-Vásquez et al. 2015). Both endogenous and exogenous SA was evidenced to play roles in antioxidant metabolism and have a tight control over cellular ROS (Kang et al. 2014). In the current scenario, the plant protection provided by induction of systemic resistance is an effective and simple approach to disease management. The resistance of plants to pathogens can be enhanced by the application of various biotic and abiotic agents. However, combining different biocontrol agents with chemical inducers can significantly enhance the resistance of a plant and contribute to better control of pathogens (El-khallal 2007a, b).

Plants have developed effective defense mechanisms to survive after pathogen attack. The generation of reactive oxygen species (ROS) including superoxide anions (O_2_^−^), hydroxyl radicals (OH^−^) and hydrogen peroxide (H_2_O_2_) is the principal indication of early plant defense response encountered with the pathogen and recognized by the same (Torres et al. 2006; Lehmann et al. 2015). One of the key functions associated with ROS system is the regulation of several biological and physiological processes including growth and development of plants and mitigation of both biotic and/or abiotic stresses by acting as a key signaling intermediates (Mittler et al. 2011; Baxter et al. 2013). Moreover, these ROS intermediates have been found to be implicated in defense response of plant through a plethora of mechanistic actions which is exemplified by cross-linking reactions of lignin and proteins causing plant cell-wall reinforcement, exposure of hypersensitive response (HR), or development of SAR against the targeted pathogen and least by acting as toxic agents against either the host plant cells, or the with development of SAR, or against the pathogen through killing them or ceasing their microbial activity. These ROS molecules have also been implicated in defense signaling pathway by acting as secondary messengers in signaling mechanism leading into the expression and activation of plant defense-related genes (Shimizu et al. 2006). Fortunately, plants possess effective mechanisms for detoxification of ROS in order to protect themselves from the toxic effects. Induction of antioxidant enzymes is a major defense mechanism of plants. Different antioxidant enzymes such as superoxide dismutase (SOD), catalase (CAT), ascorbate peroxidase (APx) and guaiacol peroxidase (GPx) involved in ROS metabolism during pathogen infection. SOD catalyses the dismutation reaction of O_2_^−^ radicals to O_2_ and H_2_O_2_. H_2_O_2_ is rapidly removed by CAT and peroxidases. CAT effectively eliminates the most of the H_2_O_2_, whereas APx can scavenge H_2_O_2_ that is inaccessible for CAT because of their higher affinity towards H_2_O_2_ and their occurrence in distinctive sub cellular locations (Creissen et al. 1994; Scandalios et al. 1997).

Here, the objective of this study was to find out the integrated effect of the biocontrol agent, *T. harzianum* and chemical inducers, SA and MeJA on biochemical changes in tomato plants against *Fusarium oxysporum* f. sp. *lycopersici* which leads to elicitation of plant defense response.

## Materials and methods

### Isolation and identification of pathogen

*Fusarium oxysporum* f. sp. *lycopersici* (Fol) was isolated from diseased tomato plants showing typical wilt symptoms on potato dextrose agar (PDA) media and incubated at 25–27 °C for 5–7 days. Stock cultures of Fol were prepared and stored at 4 °C. Pathogenicity test for all the isolates of Fol was done by following Koch’s postulate. A highly virulent isolate was selected for further experiments.

### Collection of *Trichoderma* species

*Trichoderma harzianum* BHU BOT RYRL4 (NCBI Accession No—KR 856210) and *T. asperellum* BHU BOT RYRL1 (NCBI Accession No—KR 856207) were provided from RY Roy Laboratory of Mycopathology, Department of Botany, Banaras Hindu University, Varanasi, India. *T. harzianum* MTCC 936, *T. viride* MTCC 793 and *T. asperellum* MTCC 4347 were procured from Microbial Type Culture Collection (MTCC), IMTECH, Chandigarh, India. Some spp. of *Trichoderma* was also isolated from soil samples of the rhizospheric soil of tomato plants from different places of India. All the fungal cultures were maintained in tubes of Potato Dextrose Agar (PDA) slant medium supplemented with streptomycin antibiotic and stored at 4 °C for 6 months and revived thereafter.

### Evaluation of the antagonistic activity of *Trichoderma* spp.

Different strains of *Trichoderma* spp. were screened for the evaluation of antagonistic activity against the pathogen by inoculating a 5 mm mycelial plug in the Petri plates. In the same Petri dishes perpendicular to the *Trichoderma* fungal disc, a 5 mm mycelial disc of 5-day-old pathogen culture was placed 6 cm away at the opposite side of the Petri dish. Plates were incubated at 27 ± 2 °C and growth of the pathogen mycelia towards the fungal disc was observed, and percent inhibition were measured 5 days after incubation. Data were obtained for the percentage inhibition of radial growth [100 × (C − T)/C] where C = radial growth of the pathogen in control and T = radial growth of the pathogen in dual culture with antagonist (Garrett 1956). The experiment was repeated thrice.

### Inoculum preparation

For the preparation of the inoculum, spore suspensions from 7 days old cultures of *Trichoderma* spp. were flooded and mixed with glass rod using sterilized distilled water. The suspension was then filtered through cheese cloth and further diluted with distilled water to obtain a final concentration of 1 × 10^5^ spores/ml using haemocytometer. Similarly, the inoculum preparation for pathogen was also done and the final concentration was maintained to 2 × 10^5^ spores/ml.

### Plant material and growth

Tomato (*Lycopersicon esculentum* cv. Punjab chhuara) seeds were obtained from Indian Institute of Vegetable Research (Adalpura, Varanasi) and were surface-sterilized in 0.5% sodium hypochlorite solution for 20 min and grown in autoclaved soil in greenhouse under alternate 14 h light and 10 h dark cycle at 27 and 22 °C, respectively. After attaining a height of 15–20 cm, these plants were used for testing pathogenicity against pathogen and also for further experiments.

### Pathogenicity test

Pathogenicity test was carried out by inoculating healthy 15–20 cm tall plants with the spore suspension of pathogen containing 2 × 10^5^ spores/ml followed by 48 h of incubation in the moist chamber after which the plants were returned to the green house chamber. Greenhouse temperature ranged from 25 to 29 °C with alternate 14 h light and 10 h dark cycles.

### Evaluation of tomato plant growth characteristics of different *Trichoderma* strains

An in vivo assay was used to evaluate the abilities of different *Trichoderma* strains to promote tomato plant growth. Tomato seeds were coated with an aqueous suspension containing 1 × 10^5^ spores of *Trichoderma* per ml (1 ml of spore suspension/30 seeds) and then air dried in an open petri-dish overnight under a laminar flow hood. Treated tomato seeds were sown in pots containing soil autoclaved at 121 °C for 1 h on 2 successive days. Pots with untreated tomato seeds were used as controls. The pots were incubated in a greenhouse at temperature ranged from 25 to 29 °C and watered as needed. Measurements of shoot length, stem diameter, and main root length were taken after 4 weeks.

### In vitro antifungal activity assay of SA and MeJA against *F. oxysporum* f. sp. *lycopersici* and *Trichoderma* species

The effect of SA and MeJA was tested on radial growth of Fol and different strains of *Trichoderma* spp. cultured on potato dextrose agar (PDA) medium. The PDA was amended with SA and MeJA at 50, 100, 150, 200, 250 and 300 μM concentrations. Diameters of the fungal colony were measured at 7 days after inoculation with a 5 mm diameter plug of Fol and *Trichoderma* species.

### Phytotoxic assay of SA and MeJA against tomato plants

The tomato plants were sprayed with 50, 100, 150, 200, 250 and 300 μM concentrations of SA and MeJA to detect the phytotoxicity of these elicitors against tomato plants. After 7 days, the plants were assayed for their phytotoxicity.

### Treatment of plant material

When the tomato seedlings were 3–4 weeks old and 15–20 cm tall, tomato seedlings were inoculated with *T. harzianum* at 1 × 10^5^ spores/ml and/or sprayed daily with chemical inducers, SA and MeJA (200 µM) on the leaf surface every 24 h for 3 consecutive days. Then, plants were inoculated with the spore suspension of Fol (2 × 10^5^ spores/ml).

### Plant harvest and analysis

The plants were harvested and the leaves from treated and control plants at five different stages at 0, 24, 48, 72 and 96 h post inoculation with pathogen, were collected for the assessment of active concentration of H_2_O_2_, ascorbic acid, malondialdehyde (MDA). The enzymatic activities of different antioxidative enzymes including (SOD, APx, GPx and CAT) were also determined. In addition, we have also analyzed the effect of all the treatments given over the dry weight of roots and shoots. The treatments given were provided in the following combinations, *Fusarium oxysporum* f. sp. *lycopersici* + SA + *Trichoderma harzianum*, *F. oxysporum* f. sp. *lycopersici* + MeJA + *T. harzianum, F. oxysporum* f. sp. *lycopersici* + SA, *F. oxysporum* f. sp. *lycopersici* + MeJA, *F. oxysporum* f. sp. *lycopersici* + *T. harzianum*, *F. oxysporum* f. sp. *lycopersici* challenged and healthy control. For each treatment, three replicates having five seedlings per replicate were maintained.

### 3,3′-Diaminobenzidine (DAB) staining to detect H_2_O_2_ accumulation

Histochemical detection of H_2_O_2_ was done by 3,3′-diaminobenzidine (DAB) staining as described by Thordal-Christensen et al. (1997). Leaves of control and treated plants were cut with the razor blade and were immediately placed in a beaker containing 1 mg/ml 3,3′-diaminobenzidine-hydrochloric acid (DAB-HCL), adjusted to pH 5.6 with NaOH, and were incubated in a growth chamber for 8 h in the dark. This was followed by further washing and destaining in 96% boiling ethanol. Microscopic observation was made to find out the location of cells with H_2_O_2_ accumulation. The red–brown coloration was observed in the sample that shows the sites of H_2_O_2_ deposition.

### H_2_O_2_ estimation

The H_2_O_2_ produced was determined as described by Sagisaka (1976). Leaf tissues from control and treated plants were homogenised in 5% cold trichloro acetic acid (TCA) and the homogenate was centrifuged at 17,000*g* for 10 min at 0 °C. The reaction mixture consisted of 1.6 ml of supernatant, 0.4 ml of 50% TCA, 0.4 ml of ferrous ammonium sulphate and 0.2 ml of potassium thiocyanate. The amount of H_2_O_2_ was measured by a calibration curve prepared with known concentrations of H_2_O_2_ by measuring the absorbance at 480 nm after 15 min of incubation.

### Measurement of cell death

Histochemical analysis was done to check the degree of cell death using Evans blue staining. For this, the control and treated leaves were boiled for 1 min in a freshly prepared solution of phenol, lactic acid, glycerol and distilled water (1:1:1:1) containing 20 mg/ml Evans blue. The tissues after boiling were cleared overnight in a solution of 2.5 g/ml chloral hydrate in water. This staining easily demarcates the dead cells having intense blue stain as compared to the control plants that remained unstained.

The extent of cell death was measured by the method by Baker and Mock (1994). In this method, the samples from both control and treated leaf tissues were treated separately with 1 ml 0.25% solution of Evans blue and the samples were further incubated on a platform shaker at 27 °C and 80 rpm for 20 min. The samples were washed with deionized water in order to remove the traces of the dye from leaf tissues. For the removal of tissues bound dye, the samples were transferred to a 1.5 µl centrifuge tube and treated with 1.5% aqueous sodium dodecyl sulphate (SDS). The samples were crushed with mortar and pestle and the homogenates thus obtained were then diluted with 0.5% deionized water and were vortexed for 30 s. The samples were further transferred to the fresh tubes and centrifuged at 9000*g* for 3 min. The supernatant was taken in 0.8 ml aliquot and optical density was measured spectrophotometrically at 600 nm.

### Ascorbic acid (AsA) content

Ascorbic acid was estimated by the method described by Keller and Schwager (1977). 0.1 g of fresh leaves from each of the treatments were homogenised with 2.0 ml of extracting solution (5 g oxalic acid + 0.75 g EDTA in 1000 ml of distilled water), centrifuged at 10,000*g* for 10 min and the supernatant was collected. To 1 ml of supernatant, 5 ml of 20 µg/ml of 2,6-dichlorophenol-indophenol (DCPIP) dye was added to develop color. The absorbance of the mixture was noted at 520 nm against the blank. The standard curve was prepared by using the different concentration of ascorbic acid by following the same method. The concentration of ascorbic acid is calculated by using the method of Keller and Schwager (1977).

### Lipid peroxidation (LPO)

The extent of LPO was determined by using the method as described by Ohkawa et al. (1979). Estimation of total malondialdehyde (MDA), content produced as a secondary byproduct of polyunsaturated fatty acid peroxidation by the thiobarbituric acid (TBA) reaction is a major determinant of the extent of LPO content. The leaf samples from each of the treatments were homogenized in a buffer, centrifuged and the supernatant was used as extract for the assay. The reaction mixture consisted of 0.1 ml of extract, 0.2 ml of 8.1% SDS, 1.5 ml of 20% acetic acid and 1.5 ml of 0.8% aqueous solution of TBA. The volume of the reaction mixture was maintained up to 4.0 ml with distilled water and incubated at 95 °C for 1 h in water bath. After cooling, 1.0 ml of distilled water and 5.0 ml of mixture of *n*-butanol and pyridine (15:1 by vol) was added and centrifuged at 10,000*g* for 15 min. The spectrophotometric absorbance was measured at 532 nm and the amount of MDA was calculated based on an extinction coefficient of 155 mM^−1^ cm^−1^ and expressed as nmol MDA g^−1^ FW.

### Antioxidant enzyme assay

#### Superoxide dismutase (SOD) activity

SOD (EC 1.15.1.1) activity was determined using a method described by Fridovich (1974). Leaf samples (0.1 g) from control and treated plants were homogenised in 2.0 ml of extraction buffer (0.1 M phosphate buffer containing 0.5 mM EDTA at pH 7.5) and centrifuged at 12,000*g* for 15 min at 4 °C. The supernatants thus obtained were used as enzyme extract. The reaction mixture consisted of 200 mM methionine, 2.25 mM NBT, 3 mM EDTA, 100 mM phosphate buffer (pH 7.8), 1.5 M sodium carbonate and enzyme extract. The final volume of the reaction mixture was maintained up to 3 ml. The reaction was initiated by adding 400 µl of riboflavin (2 µM) and the tubes were illuminated for 15 min. The reaction was stopped by keeping the tubes in dark. The absorbance was measured at 560 nm by measuring the ability of enzyme extract to inhibit photochemical reduction of nitroblue tetrazolium (NBT) chloride.

#### Ascorbate peroxidase (APx) activity

APx (E.C. 1.11.1.11) activity was estimated according to the method given by Nakano and Asada (1981) by measuring the oxidation of ascorbic acid at 290 nm with slight modifications. About 0.1 g leaf tissue were homogenised in 2 ml of extraction buffer (90 mM Na_2_HPO_4_ buffer, at pH 7.8, 8% glycerol, 1 mM EDTA, and 5 mM ascorbate). PVP (0.3 g/g tissue) was added and the homogenate was centrifuged at 15,000*g* for 10 min at 4 °C. The resulting supernatants were used for the enzymatic assay. Enzyme extract (200 µl) was added to the reaction mixture of 25 mM phosphate buffer (pH 7.0), 0.1 mM EDTA, 0.25 mM ascorbic acid and 1.0 mM H_2_O_2_ and the decrease in absorbance was recorded 30 s after addition of the enzyme extract. APx activity was calculated based on an extinction coefficient 2.8 mM^−1^ cm^−1^ and the enzymatic activity was expressed as nmol ascorbate oxidized min^−1^ mg^−1^ protein.

### Catalase (CAT) activity

CAT (EC 1.11.1.6) activity was determined by using the method described by Aebi (1984). Plant tissue (0.1 g) from control and treated plants were homogenised in 50 mM Tris HCl buffer (pH 8.0) containing 0.5 mM EDTA, 2% w/v polyvinylpyrrolidone, and 0.5% (v/v) Triton X100. The homogenate was centrifuged at 12,000*g* for 15 min at 4 °C, and the resulting supernatant was used as enzyme extract. The reaction mixture consisted of 300 µM phosphate buffer (pH 7.2) and 100 µM H_2_O_2_ in 1 ml enzyme extract. Activity was determined by recording O_2_ released from enzymatic dissociation of H_2_O_2_ in darkness for 1 min. O_2_ produced by the enzymatic reaction was estimated by measuring the decrease in H_2_O_2_ absorption at 240 nm (extinction coefficient of H_2_O_2_ is 0.036 mM^−1^ cm^−1^) and enzyme activity was expressed as µM H_2_O_2_ oxidized min^−1^ g^−1^ FW.

### Guaiacol peroxidase (GPx) activity

GPx (EC 1.11.1.7) activity was spectrophotometrically determined as described by Zheng and Van Huystee (1992). The oxidation of guaiacol to tetra guaiacol was estimated by the increase in absorbance at 470 nm in a reaction mixture that contained 10 mM sodium phosphate (pH 6.0), 0.3% (v/v) H_2_O_2_, 1% (v/v) tetraguaiacol, and 0.3 ml enzyme extract. The reaction was started by the addition of H_2_O_2_. The enzymatic activity was determined in terms of U/mg protein as can be extrapolated by the linear portion of the activity curve whose absorbance was measured at 470 nm. The amount of the enzyme catalysing the oxidation of 1 µmol of guaiacol min^1^ represents one unit of enzyme activity. Protein was estimated following the method of Lowry and others (1951).

### Statistical analysis

All statistical analysis was done by using SPSS ver. 16. The results are the mean of the three replicates of each of the experiment. The data were statistically analysed by using one-way analysis of variance (ANOVA) and mean separations were compared with Duncan’s multiple range tests at the p ≤ 0.05 significance level. Differences at p ≤ 0.05 were considered to be significant.

## Results

### Analysis of the antagonistic activity of *Trichoderma* species against Fol

Among the different species of *Trichoderma*, *T. harzianum* BHU-BOT-RYRL4 showed the highest percentage of inhibition of pathogen growth (83.17%). Whereas, *T. asperellum* BHU-BOT-RYRL1 showed the lowest inhibition (39.98%). *T. harzianum* MTCC 936, *T. viride* MTCC 793 and *T. asperellum* MTCC 4347 inhibited the growth of pathogen by 72.13, 68.12 and 50.27%, respectively.

### Effect of the different *Trichoderma* strains on the growth characteristics of tomato plants

All the tested strains of *Trichoderma* had a significant effect on the shoot length, root length and stem diameter of tomato plants in comparison to control (Table 1). However, among the different strains of *Trichoderma*, *T. harzianum* BHU BOT RYRL 4 showed the highest tomato plant growth characteristics viz. shoot length (147.89%) root length (192.09%) and stem diameter (147.5%) followed by *T. harzianum* MTCC 936 (shoot length; 136.76%, root length; 184.38%, stem diameter; 141.25%) in comparison to control (Table 1). Whereas, *T. asperellum* BHU BOT RYRL 1 showed the lowest plant growth characteristics (shoot length; 115.54%, root length; 123.72%, stem diameter; 111.25%).

**Table 1:** Effect of the different strains of *Trichoderma* on the plant growth characteristics of tomato

*Trichoderma* isolates	Plant growth characteristics of tomato		
	Shoot length (cm)	Stem diameter (mm)	Root length (cm)
Control	19.23^a^	0.80^a^	8.26^a^
*T. harzianum* BHU BOT RYRL 4 (NCBI Accession No—KR 856210)	28.44^e^	1.18^c^	15.86^c^
*T. asperellum* BHU BOT RYRL 1 (NCBI Accession No—KR 856207)	22.22^ab^	0.89^ab^	10.22^ab^
*T. harzianum* MTCC 936	26.30^de^	1.13^b^	15.23^b^
*T. viride* MTCC 793	25.40^cd^	1.06^b^	13.60^b^
*T. asperellum* MTCC 4347	23.34^bc^	0.96^ab^	11.67^ab^

Values followed by the same letters are not significantly different at p ≤ 0.05

Based on these results, *T. harzianum* BHU-BOT-RYRL 4 was selected for further experiments.

### In vitro antifungal activity of SA and MeJA against Fol and *T. harzianum*

The mycelial growth of Fol and *T. harzianum* was not significantly affected by SA and MeJA amendment in PDA medium. Four concentrations of SA and MeJA tested, viz., 50, 100, 150 and 200 μM, were not found to inhibit mycelial growth of Fol as well as *T. harzianum* significantly as compared to control. However, beyond 200 μM, significant reductions in the growth of both the fungi were recorded.

### Phytotoxic assay of SA and MeJA against tomato plants

Four concentrations of SA and MeJA did not show any symptoms of phytotoxicity against tomato plants. Beyond 200 μM, SA and MeJA showed phytotoxicity against the tomato plants. Hence, the concentration of 200 μM for SA and MeJA was selected for spraying on tomato plants.

### Total biomass

Significant increase in growth of tomato plants in terms of dry weight of shoot and root were observed in Fol + SA + Th and Fol + MeJA + Th treated plants in comparison to pathogen challenged plants. But the maximum dry weight of shoot and root by 137.19 and 218.75% was shown by Fol + SA + Th treated plants. Plants challenged with pathogen had decreased plant root and shoot dry weight when compared with control (Table 2).

**Table 2:** Effect of the pre-treatment of *T. harzianum* and/or chemical inducers (SA and MeJA) on the changes in dry weight of shoot and root of tomato plants challenged with *Fusarium oxysporum* f. sp. *lycopersici* (Fol)

Treatments	Dry weight of shoot (g/plant)	Dry weight of root (g/plant)
Control	4.92^c^	2.37^c^
Fol	3.28^a^	0.96^a^
Fol + Th	4.13^bc^	1.61^ab^
Fol + SA	3.91^ab^	1.32^ab^
Fol + MeJA	3.72^a^	1.21^ab^
Fol + Th + SA	4.56^c^	2.10^c^
Fol + Th + MeJA	4.31^c^	1.93^bc^

Values followed by the same letters are not significantly different at p ≤ 0.05

### Localisation of H_2_O_2_ production

**Fig. 1:**
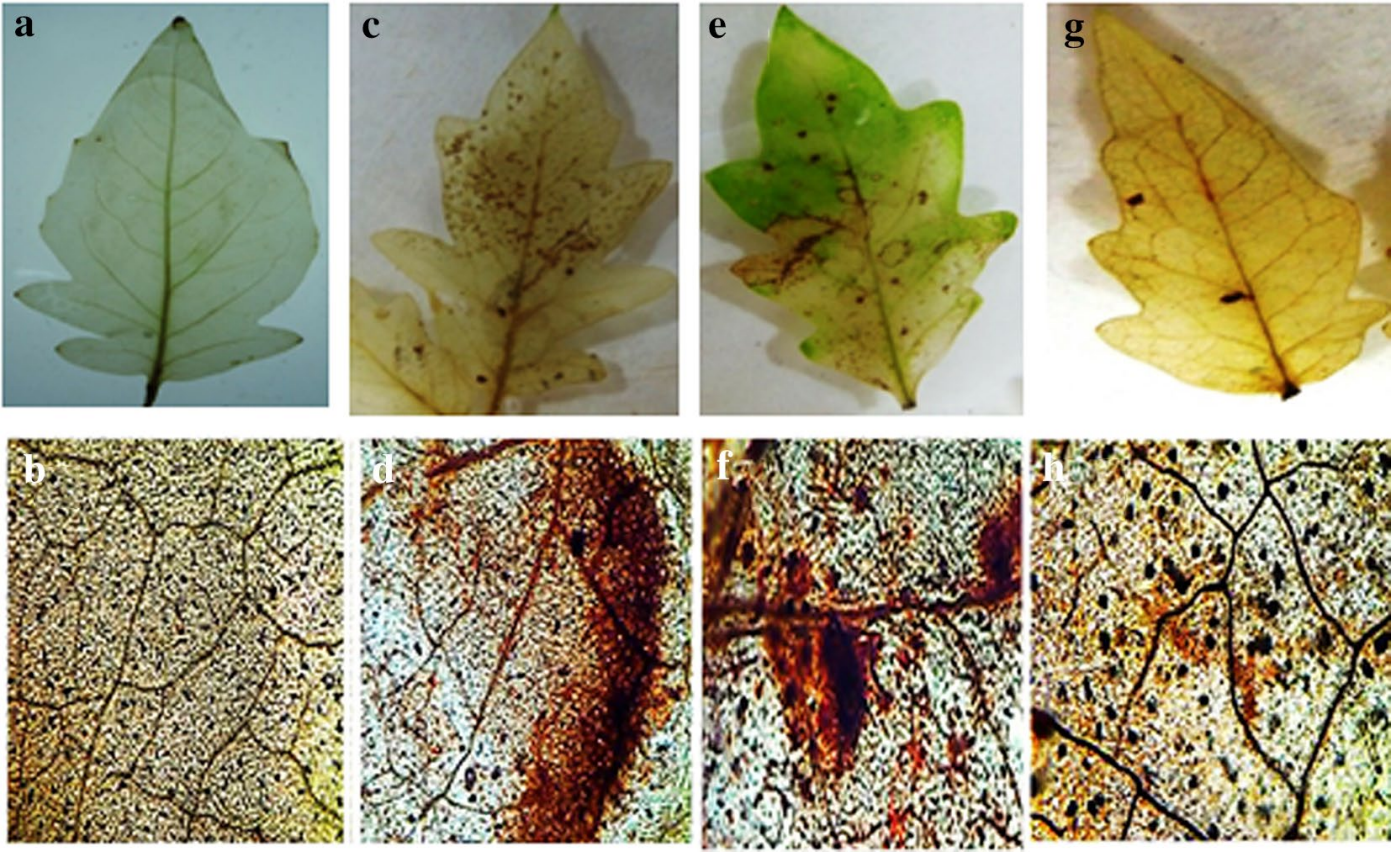
Localisation of H_2_O_2_ in different treatments of tomato leaves as revealed by reddish brown stain. **a**, **b** Unchallenged control (control), **c**, **d** challenged with pathogen (Fol). **e**, **f** Pre-treated with SA and challenge inoculated with pathogen (Fol + SA). **g**, **h** Pre-treated with *T. harzianum* (Th), MeJA and challenge inoculated with pathogen (Fol + Th + MeJA)

For the detection of potent sites generating H_2_O_2_ due to the oxidative burst, DAB staining was performed using DAB as a substrate. The principle sites of H_2_O_2_ generation among the dead cell lesions emerged with having dark brown coloration of polymerized DAB and these sites were found to be more prevalent in pathogen challenged plants followed by those plants which were pretreated with SA along with pathogen. The microscopic observation reveals that the pathogen challenged leaves showed the maximum number of cells undergoing intense dark brown coloration exposing the affected sites for H_2_O_2_ accumulation (Fig. 1d) followed by Fol + SA treated plants (Fig. 1f). Moreover, it was observed that the vascular tissues were also targeted and engaged for generating H_2_O_2_ incited by oxidative burst for those samples which were pretreated with SA along with pathogen. These dark brown coloration precipitates were totally absent from controlled untreated samples (Fig. 1a, b). In comparison between treatments, minimum induction was observed in Fol + MeJA + Th treated plants (Fig. 1g, h).

### H_2_O_2_ estimation

Treatment of tomato leaves with the pathogen, SA, MeJA and *T. harzianum* produced an oxidative burst in the form of H_2_O_2_ production. Treatment with SA especially when applied with pathogen markedly increased ROS production. Pathogen stress, SA, MeJA and Th treatment caused a sharp change in H_2_O_2_ content at 24 and 48 h. After 48 h of treatment, H_2_O_2_ decreased gradually in all treatments. Minimum H_2_O_2_ production was shown by Fol + Th + MeJA treated plants (Fig. 3a).

### Measurement of cell death

**Fig. 2:**
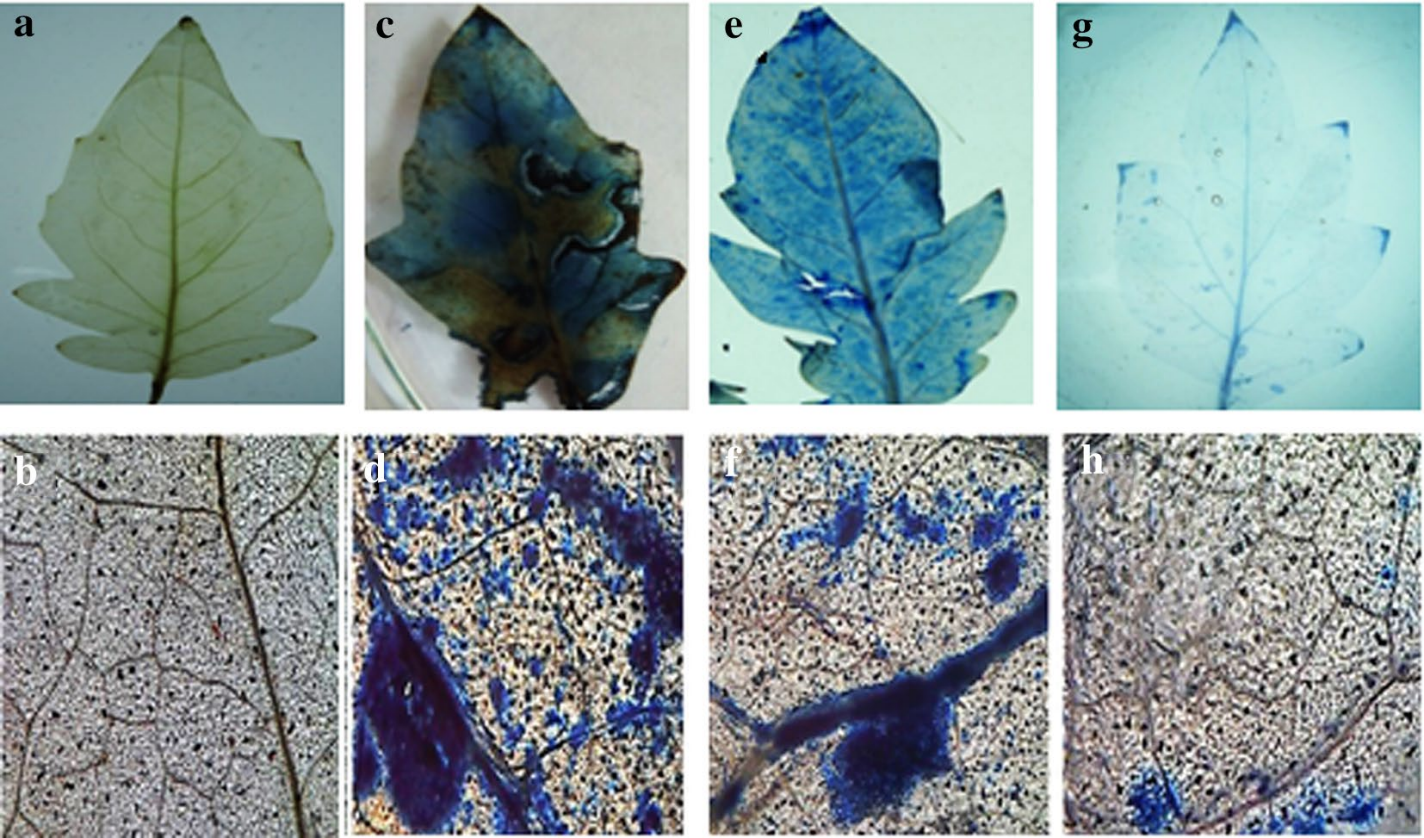
Localisation of cell death (evans blue uptake) in different treatments of tomato leaves as revealed by blue stain. **a**, **b** Unchallenged control (control), **c**, **d** challenged with pathogen (Fol). **e**, **f** Pre-treated with SA and challenge inoculated with pathogen (Fol + SA). **g**, **h** Pre-treated with *T. harzianum* (Th), MeJA and challenge inoculated with pathogen (Fol + Th + MeJA)

**Fig. 3:**
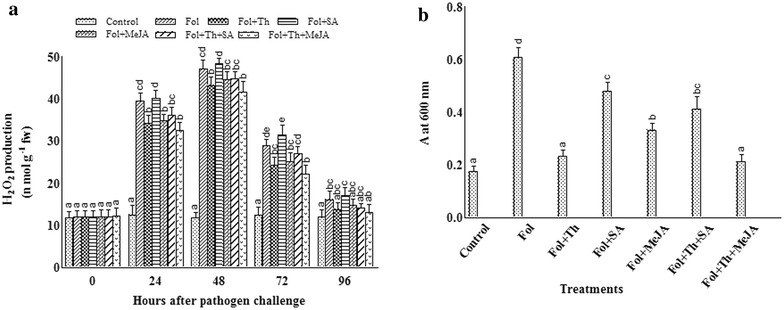
Estimation of **a** H_2_O_2_, and **b** cell death in tomato plants pre-treated with *T. harzianum*, SA and MeJA either individually or in combination after challenge inoculation with *Fusarium oxysporum* f. sp. *lycopersici* (Fol). Results are expressed as the mean of three replicates and vertical bars indicate SD of the mean. Different letters indicate significant differences among treatments within the results taken at the same time interval according to Duncan’s multiple range test at p ≤ 0.05

Microscopic examination was done after staining the cells with Evans blue dye which clearly reveals the localized region of cell death and extent of damage incited by the oxidative burst. This was easily observed and determined by examining both the treated tissues and control samples under the microscope. The variation in color intensity from light to dark blue clearly pointed out the sites of damaged tissues and the extent of their destruction profile lying in between the live cells. This observed variation in colour intensity is directly proportional to the degree of damages as the fully dead tissues take the intense blue coloration when compared to active tissues from control samples having no coloration. In this study, maximum cell death was shown in pathogen inoculated leaves followed by Fol + SA treated leaves as compared with other treatments (Figs. 2c–f, 3b). Evans blue staining showed that death did not occur in untreated leaf tissue (Fig. 2a, b) while Fol + Th + MeJA treated plants showed minimum cell death (Fig. 2g, h).

### Ascorbic acid (AsA) content

**Fig. 4:**
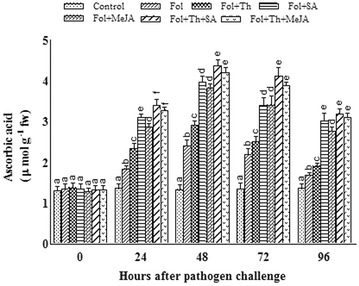
Estimation of ascorbic acid in tomato plants pre-treated with *T. harzianum*, SA and MeJA either individually or in combination after challenge inoculation with *Fusarium oxysporum* f. sp. *lycopersici* (Fol). Results are expressed as the mean of three replicates and vertical bars indicate SD of the mean. Different letters indicate significant differences among treatments within the results taken at the same time interval according to Duncan’s multiple range test at p ≤ 0.05

The level of AsA started to rise 24 h after inoculation reaching an optimal level up to 48 h and then started to decline after 72 h. The maximum AsA content was more prevalent in leaves treated with Fol + Th + SA when compared to single treatments. The total ascorbic acid was found to be highest in Fol + Th + SA treated samples whereas the control plants showed no variation in their AsA profile. AsA content was 345.6 and 176.32% higher in Fol + Th + SA treated plants as compared to control and pathogen challenged plants, respectively (Fig. 4).

### Lipid peroxidation

**Fig. 5:**
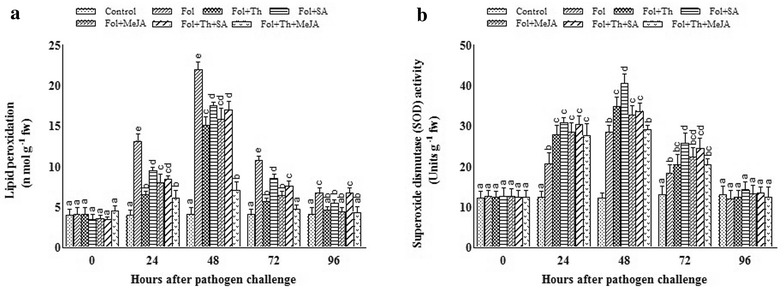
Estimation of changes in the **a** concentration of MDA **b** activity of SOD, in tomato plants pre-treated with *T. harzianum*, SA and MeJA either individually or in combination after challenge inoculation with *Fusarium oxysporum* f. sp. *lycopersici* (Fol). Results are expressed as the mean of three replicates and vertical bars indicate SD of the mean. Different letters indicate significant differences among treatments within the results taken at the same time interval according to Duncan’s multiple range test at p ≤ 0.05

The Malondialdehyde (MDA) content was significantly higher in pathogen challenged plants and was greatly reduced in the plants pretreated with Th and MeJA together as compared to the other treatments. Levels of MDA slowly increased in leaves of all the treatments but at a particularly lower level in Fol + Th + MeJA treated tomato plants as compared with the other treatments. The level of MDA gradually increased up to 48 h after pathogen infection and declined thereafter in all treatments. MDA content was 321.17% less and 188.36% higher in the Fol + Th + MeJA treated plants when compared with pathogen challenged and control plants, respectively. In Fol + Th + SA treated plants, it was 132.92% lesser than pathogen inoculated plants (Fig. 5a).

### Antioxidant enzyme activities

**Fig. 6:**
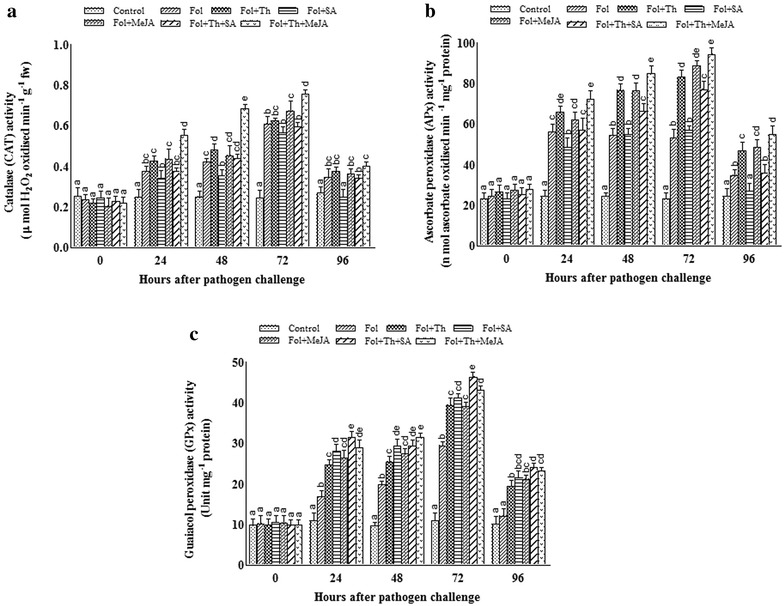
Estimation of changes in the activities of **a** CAT, **b** APx, and **c** GPx in leaves of tomato plants pre-treated with *T. harzianum*, SA and MeJA either individually or in combination after challenge inoculation with *Fusarium oxysporum* f. sp. *lycopersici* (Fol). Results are expressed as the mean of three replicates and vertical bars indicate SD of the mean. Different letters indicate significant differences among treatments within the results taken at the same time interval according to Duncan’s multiple range test at p ≤ 0.05

Infection with the pathogen resulted in the general increase in activity of antioxidant enzymes as compared to the control plants. The activities of APx, GPx and CAT increased at 24 h, peaking at 72 h and decreased slightly thereafter in all applied treatments (Fig. 6a–c). However, the SOD activity was found maximum at 48 h and declined thereafter (Fig. 5b). Treatment with chemical inducer especially SA when applied with pathogen markedly decreased H_2_O_2_ scavenging enzymes (APx and CAT) and greatly increased SOD activity in comparison with other treatments. Pre-treatment of tomato plants with Th and MeJA markedly induced H_2_O_2_ scavenging enzymes and the highest induction was recorded when applied together. The activities of APx and CAT in Fol + Th + MeJA treated plants were 173.6 and 130.87% greater than those of the pathogen inoculated plants whereas in the case of Fol + Th + SA treated plants, the activities of GPx and SOD were 168.09 and 145.13% higher than pathogen challenged plants respectively which declined thereafter.

## Discussion

ROS is speculated as the first line of active plant defense against various invading pathogens and may directly act as an antimicrobial agent (Malolepsza and Rozalska 2005; Torres et al. 2006; Shetty et al. 2007). Many reports have shown that H_2_O_2_ is a signaling molecule that plays a significant role in plant defense response (Neuenschwander et al. 1995; Orozco-Cárdenas et al. 2001). ROS generation leads to cellular damage and ultimately cell death. The estimation of cell death and degree of damages due to ROS system can be easily visualized by Evans blue dye whereas the DAB staining predicts the potent sites of H_2_O_2_ generation (Faoro and Iriti 2005). In our study, staining with DAB and Evans blue, which can visualise H_2_O_2_ accumulation and cell death demonstrates that the pathogen challenged followed by Fol + SA treated plants induced maximum H_2_O_2_ accumulation and cell death in leaf tissues in comparison to other treatments. In the present study, it was evident from the results that there was a more or less steady increase or decrease in the level of the H_2_O_2_ in all the treatments. Maximum H_2_O_2_ production was shown by the plants which were treated with SA along with pathogen or in combination with *T. harzianum,* while minimum production was shown by the Fol + Th + MeJA treated plants. Suppression of H_2_O_2_ production in leaves of Fol + Th + MeJA treatments could be associated with the increased activity of antioxidant enzymes which decreased levels of H_2_O_2_ (either by direct decomposition or oxidation). SA has been shown to increase accumulation of H_2_O_2_ in plants after pathogen infection which activates defense related genes by acting as a secondary messenger (Kauss and Jeblick 1996; Garreton et al. 2002; Anand et al. 2008; Lee et al. 2010; Khokon et al. 2011; Miura and Tada 2014). The remarkable role of SA in plants is found to be associated with the activation of many genes whose expression terminates into plant defense activities including generation of H_2_O_2_ and induction of cell death in response to fungal elicitors and wounds (Kauss et al. 1992a, b; Shirasu et al. 1997; Mur et al. 2006). On the contrary, SA is considered to play an important role in preventing the damage to plants against oxidative stress by reducing the accumulation of ROS (Yang et al. 2004). An ambivalent outcome of SA as a prooxidant and antioxidant has been shown in many stress models against various pathogens and several abiotic stresses such as high light intensity, drought, salinity, and cold (Mou et al. 2003; Mateo et al. 2006; Miura and Tada 2014).

The antioxidant enzymes play a defensive role in plants against many oxidative stresses (Saed-Moucheshi et al. 2014). It has been shown that H_2_O_2_ enhances the antioxidant capacity of cells by increasing the activity of various antioxidant enzymes such as CAT, APx and SOD (Xia et al. 2009). The results showed that in the plants infected with *F*. *oxysporum* f. sp. *lycopersici*, the activities of APx, CAT and GPx significantly increased at 48 and 72 h in comparison to control, but the highest activity of these enzymes were found in plants treated with MeJA and *T. harzianum* together. However, the plants treated with the chemical inducer SA especially when applied with pathogen, noticeably decreased H_2_O_2_ scavenging enzymes (APx and CAT) and greatly increased SOD activity. The increased SOD activity due to SA might protect biomolecules from being attacked by superoxide radicals (Belkadhi et al. 2013). The increased antioxidant enzyme activities in the MeJA and *T. harzianum* treated plants were more pronounced and higher levels were observed, indicating that the MeJA and Th pretreated plants were highly efficient in scavenging from the deleterious effects of ROS. The SOD enzyme of defense system dismutate the superoxide radicals generated after oxidative metabolism into H_2_O_2_ and O_2_ which acts as a first line of defense response in plants (Gratão et al. 2012; Radwan 2012). SA plays an important role in generation of H_2_O_2_ (by inhibiting the expression of genes encoding for APx and catalases) which can be correlated with the activation of defense related genes and other enzymes such as SA-insensitive guaiacol peroxidases having an important role in other defense related responses including lignification and crosslinking of cell wall proteins (Gillham and Dodge 1987; Rajan and Murugan 2010). Chen et al. (1993) reported that SA inhibits the catalytic activity of enzyme catalase by binding to it. It has also been proved in tobacco where SA inhibits catalase activity (Conrath et al. 1995). Similarly, the in vitro inhibitory action of SA catalase-inhibiting effect has also been demonstrated in many other plant species such as *Arabidopsis*, tomato, cucumber (Sanchez-Casas and Klessig 1994), and maize (Horváth et al. 2002).

An initial increase in ascorbic acid content was observed in our study, which was significantly higher in Fol + Th + SA treated plants when compared with pathogen challenged and healthy controls. Thereafter, a marked decline in ascorbic acid content was observed in all treatments. The initial increase in ascorbic acid content in the present study can be explained by the fact that ascorbic acid along with glutathione are present at high concentrations in chloroplast and other cellular organelles which play a promising role in redox buffering of plant cells where they play a key role in plant defense.

The free fatty acids generated after degradation of membrane lipids act as substrate for enzyme lipoxygenase (LOX), causing oxidative deterioration resulting into membrane peroxidation. The membrane peroxidation finally produces various sorts of alkoxy and peroxy radicals including singlet oxygen. These biochemical reactions occurring inside the membrane lead into the major source of ROS generation for plant cells (Farmer and Mueller 2013). In our study, the levels of MDA gradually increased in leaves of infected tomato plants with increasing the time of infection. Tomato plants which were pretreated with Th and chemical inducers (SA and MeJA), MDA levels markedly decreased as compared with pathogen challenged plants. Among these treatments, pathogen challenged plants had the highest MDA level followed by Fol + SA treated plants. The catalytic activity of SOD is associated with the extent of lipid peroxidation as evidenced from the fact that SA treated plants results into the lower degree of membrane damage when compared to plants those were inoculated with the pathogen. One of the critical function of SA involves the activation of SOD that helps in the deactivation of lipid peroxidation thus facilitates in maintaining the integrity of membrane structures of root cells (Zenkov et al. 2001; Belkadhi et al. 2013). Minimum lipid peroxidation was found in Fol + Th + MeJA treated plants. The reduction in lipid peroxidation in tomato plants treated with Th along with MeJA might be related to the enhanced activity of antioxidant enzymes preventing formation and accumulation of free radicals, and subsequently membrane damage.

The individual treatment with chemical inducers like SA or MeJA or Fol elicitors did not have major effect on defense gene expression. However, pre-treatment of plants with these chemical inducers followed by pathogenic inoculation leads into aggravated defense response and characterized by increased expression of defense related genes such as Phenylalanine ammonia lyase (*PAL*) (in case of SA treatment) or increased expression of *LOX* genes (MeJA treatment). The two different chemical inducers have different effect on plant defense gene expression in which SA works through systemic acquired resistance (SAR) pathway. The SA in this SAR mediated defense may go through direct activation of PR genes expression or in low doses do not activate defense genes directly but prime the tissue for potentiated defense-gene expression upon subsequent Fol infection. SA-mediated transcriptional responses control the temporal patterns of gene expression in response to stress. Moreover, it has been well demonstrated that SA plays crucial role in *Trichoderma* root early colonization as revealed through expression studies (Alonso-Ramírez et al. 2014). In contrast, the MeJA as well as *Trichoderma* inoculated plants mediated defense response through ISR pathways involving ethylene and other hormones. This was confirmed through mutant studies where *Trichoderma harzianum* was inoculated with wild type as well mutants affected in the biosynthesis of specific defense-related hormones were selected, including the JA-deficient *defenseless1* (*def1*), the SA-deficient *NahG*, the ABA-deficient *sitiens* and the ET-under producing ACC deaminase *ACD* (Martínez-Medina et al. 2013). In all cases the mutant plants were found to be susceptible for disease development even after *Trichoderma* inoculation, indicating that JA-regulated pathway is required for TISR against pathogen challenged conditions. Recently, Rubio et al. (2017) have evaluated the defense gene expression following the combined treatment of *Trichoderma harzianum* T34 with NPK supplemented and under salt stressed conditions and demonstrated the expression of expression of eight genes out of nine analyzed: *EIN2*, encoding a central component of the ET signaling pathway; *NPR1*, which encodes a key transducer of SA signaling involved in plant defense responses; *AREB2*, encoding a transcription factor (TF) of ABA signaling; *LERBOH1*, involved in ROS production; *APX1*, encoding an ascorbate peroxidase; *SOS1*, which encodes a salt tolerance marker; *ARF1*, encoding a TF that binds to auxin response elements; and *DREB3*, a marker gene involved in tolerance to drought. AsA is a very important antioxidant, which protects the plants under oxidative stress (Shafiq et al. 2014). It has been reported that exogenous application of JA increases AsA in different plants suggesting that JA may regulate AsA metabolism (Ayala-Zavala et al. 2010; Chen and Gu 2014).

*Trichoderma* induced systemic resistance (TISR) in tomato against Fol challenged conditions is mainly based on boosted JA-dependent responses, the pathways regulated by the plant hormones-SA is also required for successful TISR development (Martínez-Medina et al. 2013). Priming for enhanced resistance to biotic and abiotic stress obviously is operating via various pathways involved in different metabolic processes. This suggests that the mode of action of priming and the resulting potentiation of cellular defense responses rather than the direct upregulation of defense signaling cascades might be of great advantage for living organisms. Plants pretreated with bioagent and chemical inducers together become primed to respond faster and show stronger activation of cellular defense responses after pathogen challenge compared with control plants. Higher induction of antioxidant enzymes in the plants which are pretreated with biological and chemical inducers in the present study can be correlated as a defense response triggered against *F. oxysporum* f. sp. *lycopersici* invasion in tomato plants.

## Conclusion

The present study showed that the pre-treatment of the tomato plants with chemical inducers SA, MeJA and biocontrol agent *T. harzianum* induced the defense system against *F. oxysporum* f. sp. *lycopersici* infection with the increased expression of various antioxidant enzymes leading to enhanced defense response. In conclusion, it can be said that combinations of biocontrol agent and chemical inducers can be a useful and better promising measures for suppressing Fusarium wilt disease more efficiently than using alone due to the involvement of a number of defense mechanisms.

## Abbreviations

Fol: *Fusarium oxysporum* f. sp. *lycopersici*
Th: *Trichoderma harzianum*
SA: salicylic acid
MeJA: methyl jasmonate
MDA: malondialdehyde
CAT: catalase
SOD: superoxide dismutase
APx: ascorbate peroxidase
GPx: guaiacol peroxidase
ROS: reactive oxygen species
H_2_O_2_: hydrogen peroxide
SAR: systemic acquired resistance
ISR: induced systemic resistance
